# The Guiding Symptoms of Our Materia Medica

**Published:** 1892-03

**Authors:** 


THE GUIDING SYMPTOMS OF OUR MATERIA MEDICA. By C. Hering, M. D. Volume X, Philadelphia: published by the estate of Constantine Hering, 112 and 114 North Twelfth Street.

This the tenth and last volume of Hering's great work, the work that he accounted his monument, is now finished and published. The editor of this Journal has uniformly and persistently urged the immense value of this work upon the profession not alone in these pages, but in private letters to contributors and subscribers. He now repeats that no good Hahnemannian prescriber can afford to be without it. The clear arrangement in chapters of the various regions of the body, the placing of each symptom in a paragraph by itself, and the clear large type all serve to enhance its value and to save time in seeking out any given symptom. It may be ordered of the F. A. Davis Company, the well-known publishers, 1231 Filbert Street, Philadelphia, or of their house in London, England. W. M. J.

